# The effect of carbohydrate and marine peptide hydrolysate co-ingestion on endurance exercise metabolism and performance

**DOI:** 10.1186/1550-2783-10-29

**Published:** 2013-05-31

**Authors:** Jason C Siegler, Richard Page, Mark Turner, Nigel Mitchell, Adrian W Midgely

**Affiliations:** 1 School of Science & Health, University of Western Sydney, Locked Bag 1797, Sydney, NSW 2751, Australia; 2 Department of Sport, Health & Exercise Science, University of Hull, Hull, UK; 3 School of Sport, Exercise & Health Sciences, University of Loughborough, Loughborough, UK; 4 Head of Nutrition, British Cycling, Manchester, UK; 5 Department of Sport & Physical Activity, Edge Hill University, Ormskirk, UK

**Keywords:** Marine peptide, Hydrolyzed protein, Exercise metabolism

## Abstract

**Background:**

The purpose of this study was to examine the efficacy of introducing a fish protein hydrolysate (PEP) concurrently with carbohydrate (CHO) and whey protein (PRO) on endurance exercise metabolism and performance.

**Methods:**

In a randomised, double blind crossover design, 12 male volunteers completed an initial familiarisation followed by three experimental trials. The trials consisted of a 90 min cycle task corresponding to 50% of predetermined maximum power output, followed by a 5 km time trial (TT). At 15 min intervals during the 90 min cycle task, participants consumed 180 ml of CHO (67 g^.^hr^-1^ of maltodextrin), CHO-PRO (53.1 g^.^hr of CHO, 13.6 g^.^hr^-1^ of whey protein) or CHO-PRO-PEP (53.1 g^.^hr^-1^ of CHO, 11 g^.^hr^-1^ of whey protein and 2.4 g^.^hr^-1^of hydrolyzed marine peptides).

**Results and conclusions:**

During the 90 min cycle task, the respiratory exchange ratio (RER) in the CHO-PRO condition was significantly higher than CHO (p < 0.001) and CHO-PRO-PEP (p < 0.001). Additionally, mean heart rate for the CHO condition was significantly lower than that for CHO-PRO (p = 0.021). Time-to-complete the 5 km TT was not significantly different between conditions (m ± SD: 456 ± 16, 456 ± 18 and 455 ± 21 sec for CHO, CHO-PRO and CHO-PRO-PEP respectively, p = 0.98). Although the addition of hydrolyzed marine peptides appeared to influence metabolism during endurance exercise in the current study, it did not provide an ergogenic benefit as assessed by 5 km TT performance.

## Background

The ergogenic effects of carbohydrate (CHO) feedings during endurance exercise are well established [1,2]. Recently, a number of studies have proposed that the addition of protein to a CHO solution (CHO-PRO) may further augment exercise performance beyond that of CHO supplementation alone [3-5]. However, evidence of performance enhancement remains equivocal, with others observing no additional benefits [6-10] and even ergolytic effects [11]. The discrepant findings may be methodological and based largely upon both variations in CHO feeding strategies [1-4,12] and caloric content of various protein solutions [3-5]. However, and in specific reference to those studies reporting an ergogenic effect, it is unclear whether the reported benefits were mediated by a protein-specific mechanism or simply the additional energy content provided within the CHO-PRO treatments [13].

Another potential mediating factor receiving less attention in the literature may be the influence of different protein sources [13,14], as a majority of studies to date have used only whey protein [14]. Recently, a small body of research has emerged exploring the potential benefit of co-ingesting protein hydrolysates with CHO during endurance exercise [13,15]. Protein hydrolysates are produced from purified protein sources, with each hydrolysate being a mixture of various length peptides together with free amino acids. Hydrolysates consisting of small chain amino acids have been shown to increase digestion and absorption kinetics [16,17] and induce a greater insulinemic response when ingested alone [17] or with CHO post exercise [18,19]. However protein hydrolysates differ from one another nutritionally, and may therefore elicit different physiological responses [20]. For example, chronic consumption of hydrolysates produced from fish protein has been shown to increase fatty acid oxidation and reduce adipose tissue mass in rats when compared to an equal energetic amount of soy protein [21].

The increased reliance on lipid metabolism observed by Liaset and colleagues has provided the rationale for others to explore the potential performance enhancing effects of fish protein hydrolysates in the context of endurance exercise in humans. The novel work of Vegge and colleagues aimed to determine if a commercially available fish protein hydrolysate (Nutripeptin™) would improve endurance capacity better than either CHO or CHO plus whey protein consumption [15]. The results did not substantiate a performance benefit *per se* (as assessed at the end of the endurance ride with a five minute mean-power test), however the authors did observe similar physiologic responses between the carbohydrate and Nutripeptin™ conditions, but not the carbohydrate plus whey condition. Although these findings were inconclusive, the positive performance response of some participants and the evidence suggesting there may be a metabolic influence (i.e. greater fat oxidation) warrants further investigation. Therefore, the purpose of the current study was to further examine the efficacy of introducing a fish protein hydrolysate concurrently with CHO and whey protein on endurance exercise metabolism and performance.

## Methods

### Subjects

Twelve apparently healthy men volunteered to participate in the study and had the following characteristics: median (IQR) age of 23 (6) years; height (mean ± SD) 176.5 ± 5.7 cm; body mass 76.0 ± 8.3 kg; maximal oxygen consumption (VO_2max_) 52.5 ± 5.2 ml^.^kg^.^min^-1^; and maximal power output (W_max_) 294 ± 19 W. All were engaged in aerobic training 3–5 d^.^wk^-1^ prior to and throughout the data collection period. The investigation was approved by the local institution’s Human Research Ethics Committee and was conducted in accordance with the Declaration of Helsinki.

Participants were instructed to maintain their habitual dietary and fluid intake prior to both the familiarisation and experimental trials. All participants were provided with a food diary to record food and fluids consumed 24 hours prior to entering the laboratory, and in order to replicate dietary intake for subsequent trials. Participants were also instructed to abstain from alcohol and caffeine for 24 hours prior to all visits and none were known to be consuming any prescription medications, or other ergogenic substances that may have affected energy transfer [22]. Participants were instructed to maintain the same training frequency, volume and intensity at the initiation of the study for the duration of the investigation, but to refrain from exercise during the 24 hours prior to entering the laboratory.

### Experimental protocol

The study followed a randomised, double blind crossover design. Initial testing consisted of an assessment of maximal oxygen uptake (VO_2_max) and maximal power output (W_max_) utilizing an incremental cycle test to exhaustion. Participants then returned to the laboratory on a further four occasions (7–10 days apart) to complete firstly a familiarisation and subsequently the experimental trials. All trials consisted of a 90 minute (min) cycle task at 50% W_max_ followed by a 5 km time trial. Participants arrived at the laboratory approximately 12 hours post prandial and all testing was initiated at 0900 to minimize any influence of circadian variation. All procedures were conducted at sea level in a thermo-neutral laboratory environment (temperature: 21.0 ± 1.2°C; humidity: 40 ± 6 %; barometric pressure: 761 ± 8 mmHg).

### Maximal oxygen consumption & maximal power output assessment

During their initial visit to the laboratory, body mass (SECA digital weighing scales, SECA, Birmingham, UK) and height (Holtain stadiometer, Holtain, Crymych, Dyfed) were recorded prior to testing along with each participant’s desired ergometer orientation, which was replicated during subsequent visits. VO_2max_ and W_max_ were determined utilizing a step-incremented protocol to exhaustion on an electromagnetically braked cycle ergometer (Lode Sport Excalibur, Lode B.V. Medical Technology, Groningen, Netherlands) and following the methods of Currell and Jeukendrup [23]. Briefly, the protocol consisted of a three minute warm-up at 95 W proceeded by an increase of 35 W every three minutes until fatigue with the ergometer set in cadence independent (hyperbolic) mode [23]. Pulmonary oxygen uptake (VO_2_), carbon dioxide production (VCO_2_) and respiratory exchange ratio (RER) were determined continuously during exercise via an automated metabolic gas analyzer (Cortex Metalyzer 3B-R2, Cortex Biophysic, Leipzig, Germany). The modular gas analyzers were calibrated with gases of known concentrations (17.05% O_2_, 4.98% CO_2_, Cranlea, Birmingham, UK) and ambient air. The volume sensor was calibrated with a 3 L calibration syringe (Hans Rudolph model 5530, Hans Rudolph, Kansas, USA). Heart rate was recorded continuously using a heart rate monitor (Polar, Polar Electro, OY, Finland). The highest 11-breath rolling average (centered to the middle breath) was considered to be VO_2max_[24]. This value was considered maximal with a plateau in VO_2_ (< 2 ml^.^kg^.^min^-1^) with increasing test duration/work rate. In the absence of a discernible plateau secondary criteria, which included 1) heart rate within 10 beats^.^min^-1^ of age predicted maximum heart rate (220 - age), 2) RER > 1.10 and 3) RPE > 17 were utilized. Maximum power output was calculated from the power output during the last completed stage, plus the fraction of time spent in the final non-completed stage multiplied by the work rate increment (i.e. W_max_ = W_com_ + [t/180] × 35, where W_com_ is the power output during the last completed stage, t is the time in seconds spent in the final non-completed stage and 35 is the work rate increment in watts) [23]. These values were then used to determine the power output for the 90 min cycle task corresponding to 50% W_max_.

### Familiarization & experimental trials

During their second visit to the laboratory, participants performed a familiarisation trial consuming water only following the identical feeding strategy to that of the actual treatment beverages. All pre-trial and trial conditions were replicated for the subsequent three experimental trials. Participants arrived at the laboratory approximately 12 hours postprandial and had been instructed to consume 500 ml of water before bed and the same volume again on waking to ensure they were adequately hydrated. Upon arrival a urine sample was initially obtained and assessed for osmolality (Osmometer, Advanced Instruments Model 3320, Advanced Instruments Inc., Massachusetts, USA). Each individual’s body mass was then recorded with participants wearing shorts only and repeated again post exercise along with urine osmolality. Participants were fitted with a heart rate monitor and mounted the electromagnetically braked cycle ergometer. They then began the 90 min bout of cycling corresponding to 50% of their previously determined W_max_ (147 ± 10 W), with the cycle ergometer set in cadence independent mode. During the 90 min period capillary blood samples, HR and RPE were obtained every 15 min. Expired air (VO_2_, VCO_2_ and RER) was measured during each 10 min period between feedings (i.e. 5–15, 20–30, 35–45, 50–60, 65–75 and 80–90 min) when the oso-nasal mask was removed for a five min interval. Participants were blinded to all physiological and output data during the task.

On completion of the 90 min cycle task, participants were immediately transferred to an air-braked cycle ergometer (Wattbike, Wattbike Ltd, Nottingham, UK) to perform a 5 km time trial. The time trial began exactly one min after the termination of the 90 min cycle task. The ergometer display was covered so that participants could only view the distance remaining to completion. No other visual feedback regarding performance was provided; however, participants were given strong verbal encouragement to complete the time trial as quickly as possible.

### Blood analysis

All blood samples were obtained in duplicate aseptically from the fingertip via lancet (Accu-Chek Safe-T-Pro Plus single-use sterile lancets, Roche Diagnostics, Mannheim, Germany) and collected in 100 μL electrolyte balanced heparin coated capillary tubes (Radiometer, West Sussex, UK). Samples were immediately analyzed (95 μL) for whole blood glucose and lactate using a clinical blood gas and electrolyte analyzer (ABL 800 basic, blood gas and electrolyte analyzer, Radiometer, West Sussex, UK).

### Nutritional intervention

Participants consumed three different beverages all matched for energy content: CHO only (67 g^.^hr^-1^ of maltodextrin derived from corn starch); CHO-PRO (53.1 g^.^hr^-1^ of maltodextrin, 13.6 g^.^hr^-1^ of whey protein concentrate); or CHO-PRO-PEP (53.1 g^.^hr^-1^ of maltodextrin, 11.0 g^.^hr^-1^ of whey protein concentrate, 2.4 g^.^hr^-1^ of peptides (fish meat hydrolysate extracted from salmon)). Treatment beverages were blinded by the manufacturer and provided in powder form (Nutrimarine Life Science, Bergen, Norway). Prior to each trial the powder was weighed (Kern EW 120-4NM electronic bench-top scales, Kern & Sohn GmBH, Belingen, Germany) and subsequently mixed with water (magnetic stirrer HI-200 M, Hanna Instruments, Bedfordshire, UK) in accordance with the manufacturer’s recommendations, with the addition of 5 ml of lemon food flavoring added to each total dose (1080 ml) to enhance blinding and palatability. All solutions were administered via an opaque drinks bottle. Participants consumed 180 ml of each respective beverage every 15 min of the 90 min cycle starting at the onset of exercise.

### Statistical analysis

All statistical analyses were conducted using IBM SPSS Statistics 19 (SPSS Inc., Chicago, IL). Central tendency and dispersion of the sample data are reported as the mean and standard deviation for normally distributed data and the median and interquartile range otherwise. Comparisons of means across the three experimental conditions and time (where applicable) for all outcome variables were performed using the MIXED procedure. The factors Condition and Time were both included in the model as categorical variables for body mass, urine osmolality, time trial time, mean and peak power output and VO_2_. Time was treated as a continuous variable for heart rate, RER, blood glucose concentration, blood lactate concentration and RPE. The residuals for the urine osmolality model were positively skewed, which was corrected with natural log transformation of the observed data. Two-tailed statistical significance was accepted as p < 0.05.

## Results

### Body mass and urine osmolality

There were no significant differences between experimental conditions for body mass, (F = 0.001, p > 0.99) or urine osmolality (F = 0.03, p = 0.97) before exercise. With respect to the changes across time, body mass (F = 24.1, p < 0.001) and urine osmolality (F = 7.4, p = 0.009) significantly decreased from pre to post exercise (mean weight loss of 0.4 ± 0.1 kg; mean osmolality decrease of 111.6 ± 92.6 mOsmol^.^kg^-1^), although this effect was not moderated by experimental condition for either body mass (F = 0.9, p = 0.42) or urine osmolality (F = 0.08, p = 0.92).

### 90 min cycling task

**Figure 1:**
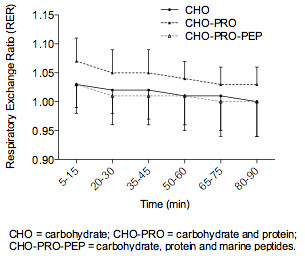
Presented are the calculated respiratory exchange ratios (RER) over the 90 minute cycling time-course of 15–20, 20–30, 35–45, 50–60, 65–75 and 80–90 minutes for each of the three experimental conditions.

Table 1 & Figure 1 indicates the mean heart rate and RER (calculated from VO_2_ & VCO_2_ data) over the 90 min constant work rate cycling bout for each of the three experimental conditions. On average, the heart rate changed by 15 bpm over the 90 min (95% CI = 11 to 19, t = 8.3, p < 0.001), which was not significantly different between conditions (F = 0.6, p = 0.58). Heart rate, however, exhibited a significant quadratic response profile (F = 14.8, p < 0.001), which was moderated by condition (F = 3.1, p = 0.048). The quadratic effect was more pronounced in the CHO-PRO condition compared to the CHO condition (t = 2.4, p = 0.015). Mean heart rate for CHO was significantly and consistently lower than in the CHO-PRO (mean difference = 4 bpm; 95% CI = 1 to 7; t = 2.5, p = 0.021). There were no significant differences between CHO and CHO-PRO-PEP (mean difference = 2 bpm; 95% CI = −1 to 5; t = 1.6, p = 0.13) and between CHO-PRO and CHO-PRO-PEP (mean difference = 1 bpm; 95% CI = −2 to 4; t = 0.9, p = 0.37).

The VO_2_ increased by approximately 0.2 L · min^-1^ over the 90 min (F = 6.1, p < 0.001), but there were no significant differences between conditions, either as a main effect (F = 0.07, p = 0.94), or as an interaction with time (F = 0.8, p = 0.67). A main effect for time was observed

 for RER (F = 14.0, p < 0.001), where the RER decreased by an average of 0.035 units over the 90 min (95% CI = 0.015 to 0.054, t = 3.4, p = 0.001) and this decrease was relatively consistent across conditions (F = 0.6, p = 0.54). The main effect for condition was statistically significant (F = 14.2, p < 0.001), where the RER in the CHO-PRO condition was consistently higher than in the CHO (mean difference = 0.028, 95% CI = 0.015 to 0.041, t = 4.2, p < 0.001) and CHO-PRO-PEP (mean difference = 0.030, 95% CI = 0.017 to 0.043, t = 4.4, p < 0.001) conditions (Figure 1). The RER in the CHO and CHO-PRO-PEP conditions were extremely similar (mean difference = 0.0015, 95% CI = −0.012 to 0.015, t = 0.2, p = 0.82, Figure 1).

Table 2 indicates the mean blood glucose, blood lactate and RPE responses over the 90 min cycling bout for each of the experimental conditions. There was a significant main effect of time for blood glucose (F = 19.7, p < 0.001), where the blood glucose decreased by an average of 0.3 mM over the 90 min (95% CI = 0.2 to 0.5, t = 4.0, p < 0.001); however, there was no significant main effect for condition (F = 0.3, p = 0.76) and no significant interaction between condition and time (F = 0.3,

**Table 1:** Heart rate (mean ± SD) in bpm over the 90 minute cycling time-course of 0–5, 15–20, 30–35, 45–50, 60–65, 75–80 and 90 minutes for each of the three experimental conditions

**Heart rate (bpm)**							
***Time (min)***	***0-5***	***15-20***	***30-35***	***45-50***	***60-65***	***75-80***	***90***
CHO	124 ± 10	128 ± 11	131 ± 9	133 ± 11	135 ± 10	137 ± 10	141 ± 12
CHO-PRO	126 ± 9	132 ± 12	136 ± 12	138 ± 12	140 ± 12	141 ± 12	142 ± 13
CHO-PRO-PEP	126 ± 11	131 ± 12	134 ± 11	137 ± 12	138 ± 12	140 ± 11	141 ±10

*CHO* carbohydrate; *CHO-PRO* carbohydrate and protein; *CHO-PRO-PEP* carbohydrate, protein and marine peptides.

**Table 2:** Blood glucose and lactate (mean ± SD) profile over the 90 minute cycling time-course of 0–5, 15–20, 30–35, 45–50, 60–65, 75–80 and 90 minutes for each of the three experimental conditions

**Blood glucose (mmol · L**^**-1**^**)**							
*Time (min)*	*0-5*	*15-20*	*30-35*	*45-50*	*60-65*	*75-80*	*90*
CHO	5.5 ± 0.6	5.6 ± 0.5	5.6 ± 0.6	5.5 ± 0.5	5.4 ± 0.4	5.3 ± 0.4	5.1 ± 0.8
CHO-PRO	5.5 ± 0.3	5.5 ± 0.4	5.5 ± 0.4	5.4 ± 0.3	5.2 ± 0.3	5.2 ± 0.3	5.3 ± 0.4
CHO-PRO-PEP	5.5 ± 0.5	5.6 ± 0.6	5.4 ± 0.8	5.4 ± 0.4	5.3 ± 0.2	5.3 ± 0.3	5.4 ± 0.2
**Blood lactate (mmol · L**^**-1**^**)**							
*Time (min)*	*0-5*	*15-20*	*30-35*	*45-50*	*60-65*	*75 -80*	*90*
CHO	2.8 ± 1.0	2.9 ± 1.3	2.5 ± 1.0	2.4 ± 0.8	2.0 ± 0.8	1.8 ± 0.4	1.9 ± 0.5
CHO-PRO	3.0 ± 0.9	3.0 ± 1.1	2.6 ± 2.3	2.3 ± 0.7	2.0 ± 0.6	1.9 ± 0.4	1.7 ± 0.3
CHO-PRO-PEP	2.9 ± 0.9	2.9 ± 1.0	2.4 ± 0.8	2.3 ± 0.8	1.9 ± 0.7	2.1 ± 0.6	2.0 ± 0.7

*CHO* carbohydrate; *CHO-PRO* carbohydrate and protein; *CHO-PRO-PEP* carbohydrate, protein and marine peptides.

 p = 0.73). There was no appreciable overall difference in blood lactate concentrations between conditions (F = 0.8, p = 0.46), however there was a significant decrease in blood lactate concentration over the 90 min (F = 27.7, p = < 0.001), which was moderated by condition (F = 4.3, p = 0.016). The blood lactate concentration decreased at a rate of 0.017 mM per min in the CHO-PRO condition, which was significantly faster than the 0.011 mM per min in the CHO-PRO-PEP condition (mean difference = 0.006, 95% CI = 0.002 to 0.009, t = 2.9, p = 0.004). No significant differences were evident between the regression slopes for CHO and CHO-PRO (mean difference = 0.0033, 95% CI = −0.00057 to 0.0071, t = 1.7, p = 0.095) and between CHO and CHO-PRO-PEP (mean difference = 0.0024, 95% CI = −0.0013 to 0.0061, t = 1.3, p = 0.21). Mean RPE significantly increased from approximately 9 to 12 units over the 90 min (F = 23.6, p = 0.001) and also exhibited a quadratic trend, where the rate of increase in RPE slowed down over time (F = 64.3, p < 0.001). The RPE was very similar across conditions, both as a main effect (F = 0.06, p = 0.94) and as an interaction with time (F = 0.3, p = 0.76).

### 5 km time trial

There were no significant mean differences between conditions for time trial time (s) (CHO: 456 ± 16; CHO-PRO: 456 ± 18; CHO-PRO-PEP: 455 ± 21; F = 0.02, p = 0.98) or mean power output (W) (CHO: 241 ± 22; CHO-PRO: 244 ± 28; CHO-PRO-PEP: 245 ± 32; F = 0.4, p = 0.67).

## Discussion

The purpose of the current investigation was to determine whether including hydrolyzed marine peptides derived from salmon meat within a CHO-PRO solution (CHO-PRO-PEP) when compared to an iso-energetic CHO only and CHO-PRO beverage effects endurance exercise metabolism. The novel findings of the study were that physiologic measures indicative of substrate utilization, such as RER, were significantly influenced according to the solution consumed during the 90 min cycle task. Heart rate was also moderated by the treatment received during this 90 min period. In contrast, no such effects (physiologic or performance) were evident during the 5 km cycling time trial.

The discrepancy between RER values during the CHO-PRO condition, compared to the CHO-PRO-PEP and CHO, warrants further clarification and discussion. At the time of the current study’s conception, the study conducted by Vegge and colleagues [15] was only available as a conference proceedings paper. As the preliminary findings indicated a potential performance enhancing effect of the protein hydrolysate, we believed further investigation was warranted. Therefore, the methodological construct of the current study was aimed at replicating the original work of the Vegge study that was presented in the conference proceedings. A secondary aim of the current study was to observe the influence of the marine peptides on the metabolic response in a more heterogeneous athletic population (refer to *Subjects* section in *Methods*). Again, this aim was derived from the findings of Vegge and colleagues, which reported a more pronounced, ergogenic effect of peptide supplementation on those athletes of *lesser* ability [15]. However, it is this secondary aim that most likely inflated the metabolic demand of the participants in the current study as evidenced in the high RER values (Figure 1) and increased cardiovascular strain during the 90 min cycle task (Table 1). We acknowledge this as a limitation in our outcome interpretations, however believe that the findings observed between experimental conditions during this potentially non steady-state 90 min cycling task further expand the limited human performance data related to hydrolyzed peptide supplementation.

As previously addressed, the differences between experimental conditions observed during the 90 min cycling task are most pronounced in the metabolic profile of the participants. RER within the CHO-PRO condition was significantly and consistently higher than that in both the CHO and CHO-PRO-PEP conditions (Figure 1). Conversely, RER within the CHO and CHO-PRO-PEP treatments exhibited very similar profiles. One plausible explanation for this discrepancy between conditions may be the influence of solution osmolality. Unfortunately we were unable to verify solution osmolality in the current study, however others have reported variations in gastric emptying rates resulting from the consumption of different forms of intact proteins [25,26]. Subsequently, exogenous CHO oxidation may have been reduced as a consequence of the delayed absorption of co-ingested CHO within the CHO-PRO condition [26,27], in which greater reliance would have been placed upon endogenous CHO reserves. In contrast, it is also possible that the inclusion of peptides within the CHO-PRO-PEP condition may have enhanced gastric emptying and gastrointestinal uptake of CHO via the up-regulation of additional intestinal co-transporters [17,28-30]. Again, however, further measurements of gut motility and absorption kinetics are required to verify the influence of solution osmolality.

The issue of solution osmolality may also be evident in the cardiovascular strain experienced by participants in the CHO-PRO condition [29]. Mean heart rate was significantly and consistently lower in the CHO compared to the CHO-PRO condition (Table 1), however no differences were apparent between the CHO and the CHO-PRO-PEP treatments. As well as affecting substrate availability, fluid may have also remained within the gastrointestinal tract and subsequently resulted in disturbances in fluid balance, reduced blood (plasma) volume and thereby potentially increased cardiovascular and thermoregulatory strain in the CHO-PRO condition [31,32]. Although direct thermoregulatory measures were not obtained in the current study, both body mass (mean weight loss of 0.4 ± 0.1 kg) and urine osmolality (111.6 ± 92.6 mOsmol^.^kg^-1^) decreased consistently across experimental conditions, which could arguably be interpreted as a consistent level of thermoregulatory strain. Additionally, and although changing at different rates, mean lactate values were not different across beverage conditions indicating that the overall glycolytic demand remained consistent between trials. As there is very little mechanistic data available on the human exercise response and peptide hydrolysate consumption, expanding further on the topic of cardiovascular strain to include potential associations between bioactive compounds and physiological control mechanisms such as angiotensin-converting enzyme (ACE) inhibition [30,33,34] at this point remains tenuous and speculative.

Regarding exercise performance as assessed via the 5 km time trial, the results of the current study are largely consistent with others who have reported no additional ergogenic effects with CHO-PRO [8-11,35] beyond that of CHO alone. There are, however, a limited number of studies that have demonstrated significant improvements in exercise capacity with simultaneous CHO-PRO supplementation [3,5]. Although in contrast to these studies, it would appear that when CHO is provided at optimal rates to produce maximal exogenous CHO oxidation (≥ 60 g^.^hr^-1^) [2], that the addition of protein [9-11] and/or protein hydrolysates [6,13,15] provide no additional ergogenic effects. Furthermore, at present no investigation utilizing ecologically valid assessments of exercise performance, as opposed to exercise capacity [36], have observed performance enhancing effects when co-ingesting protein [7,10,11] and/or protein hydrolysates with CHO [6,13,15], with which our findings are consistent. Aside from methodological issues pertaining to beverage composition and protocol design, it has been postulated that participants with a lower performance level may be more responsive to CHO-PRO-PEP supplementation than those individuals who are deemed more superior performers [15]. This notion was based on a performance factor calculated from W_max_, VO_2max_ and the mean power output from a familiarisation of a 5 min all-out cycling performance test, and a subsequent correlation analysis [15]. However, as presented previously, we did not observe an ergogenic response in our participant population.

In conclusion, the results of the present study suggest that when matching CHO, CHO-PRO and CHO-PRO-PEP solutions for energetic content, the inclusion of protein hydrolysates produced from salmon may have significant effects upon exercise metabolism during endurance cycling. However, the translation of these significant metabolic effects into subsequently meaningful performance benefits remains to be determined. Moreover, in the absence of an empirically supported mechanism, further investigations are warranted to potentially elucidate mechanisms and further determine the efficacy of CHO-PRO-PEP co-ingestion.

## Competing interests

The authors declare that they have no competing interests.

## Authors’ contributions

JS and RP were the principle investigators of the study. MT aided with data collection and analysis. JS, NM and AM conceived of the study, and participated in its design and coordination and helped to draft the manuscript. NM provided the supplements and proposed the idea of the study. All authors read and approved the final manuscript.
